# Dynamics of the Liver Stiffness Value Using Transient Elastography during the Perioperative Period in Patients with Valvular Heart Disease

**DOI:** 10.1371/journal.pone.0092795

**Published:** 2014-03-26

**Authors:** Young Eun Chon, Seung Up Kim, Jun Yong Park, Do Young Kim, Sang Hoon Ahn, Kwang-Hyub Han, Chae Yoon Chon, Sak Lee

**Affiliations:** 1 Department of Internal Medicine, Yonsei University College of Medicine, Seoul, Korea; 2 Institute of Gastroenterology, Yonsei University College of Medicine, Seoul, Korea; 3 Department of Thoracic and Cardiovascular Surgery, Yonsei University College of Medicine, Seoul, Korea; 4 Liver Cirrhosis Clinical Research Center, Seoul, Korea; University of Modena & Reggio Emilia, Italy

## Abstract

**Background/Aims:**

Liver congestion due to heart failure in patients with valvular heart disease (VHD) can result in an overestimate of the liver stiffness (LS) as assessed by transient elastography (TE). This prospective pilot study investigated the dynamics of LS during the perioperative valve operation period.

**Methods:**

Thirty-two patients who underwent a valve operation (case) and 12 who underwent a varicose vein operation (control) were prospectively enrolled. LS and cardiologic parameters at three time points [baseline, post-operative day (POD)7, and POD90] were collected.

**Results:**

LS at three time points were consistently higher in the case group than those in the control group, although all participants did not show evidence of underlying chronic liver disease (all *P*<0.05). In the case group, LS at POD7 increased slightly from the LS at baseline (median 7.9→8.4 kPa, *P* = 0.816), whereas LS at POD90 decreased significantly from the LS at POD7 (median 8.4→6.0 kPa; *P* = 0.026). LS was significantly correlated with N-terminal-pro brain natriuretic peptide (NT-proBNP) (ρ = 0.412), left ventricular ejection fraction (ρ = −0.494), and central venous pressure during the operation (ρ = 0.555) at baseline (all *P*<0.05). LS was significantly correlated with NT-proBNP (ρ = 0.526) and right ventricular pressure (ρ = 0.572) at POD7, whereas LS was significantly correlated with NT-proBNP (ρ = 0.590) at POD90 (all *P*<0.05).

**Conclusions:**

LS can be overestimated in patients with VHD due to hepatic congestion. However, LS can be dynamically reversed during the perioperative period reflecting the restoration of cardiac function after a successful operation.

## Introduction

The liver stiffness measurement using transient elastography (TE) is a rapid, noninvasive, and reproducible method of assessing liver fibrosis in patients with chronic liver diseases such as chronic hepatitis C, chronic hepatitis B, alcoholic liver disease, and primary biliary cirrhosis [1], [2]. After vigorous validation over the past several years [3], [4], TE is now being used widely in clinical practice as an excellent tool for diagnosing cirrhosis when clinical data and physical examinations are indecisive. However, the liver stiffness (LS) does not always directly reflect the degree of liver fibrosis, as it can be affected by several extrinsic conditions. Acute necroinflammation of the liver, reflected by high alanine aminotransferase (ALT) level, and cholestasis can considerably limit the accuracy of TE to assess liver fibrosis [5], [9]. Deformation of hepatic vascular architecture in liver-related sinusoidal diseases or vascular diseases can also influence TE performance [10], [11].

In addition, TE has been reported to be unreliable in patients with heart failure (HF) [12], [13]. A pathological condition of attenuated blood pumping in patients with HF causes reduced blood flow throughout the body, leading to liver congestion accompanied by sinusoidal dilation, which increases the LS. Indeed, right-sided HF elevates right ventricular pressure (RVP), which transfers directly to increased hepatic venous pressure. In the end, increased LS due to hepatic congestion leads to an overestimate of liver fibrosis and/or misdiagnosed cirrhosis. In a pilot study by Millonig *et al*. [14], the authors revealed that LS is directly influenced by central venous pressure (CVP) using landrace pigs, and insisted that elevated CVP should be considered when assessing the degree of fibrosis. However, no human studies have simultaneously demonstrated the dynamics of LS and its correlations with cardiologic parameters. In this prospective study, we demonstrated the clinical use of TE during the perioperative period in patients with valvular heart disease (VHD) by investigating the dynamics of LS and its correlation with cardiologic parameters.

## Materials and Methods

### Patients

We prospectively enrolled 32 consecutive patients with VHD (case group) from May 2010 to December 2011 who underwent valve replacement or a repair operation [mitral valve (MV) for 15 patients, tricuspid value (TV) for three patients, and both MV and TV for 14 patients, respectively]. We recruited 12 consecutive patients during the same study period who underwent a varicose vein operation (control group).

Exclusion criteria were the following: 1) history of chronic liver disease (positive for either the hepatitis B virus surface antigen, hepatitis B virus core antibody, or hepatitis C virus antibody) (*n*  =  0), 2) fatty liver on ultrasonography (either alcoholic or non-alcoholic) (*n*  =  0), 3) LS measurement failure or invalid LS (*n*  =  0), 4) withdrawal of consent (*n*  =  1 in case group), and 5) lost to follow-up (*n*  =  1 in control group). Finally, 31 patients with VHD and 11 patients with varicose veins were eligible for the statistical analysis.

This study protocol was consistent with the ethical guidelines of the 1975 Declaration of Helsinki and was approved by the institutional review board of Yonsei University Health System, Severance Hospital. A written consent form was signed by all patients.

### Endpoints and follow-up

The primary endpoint was the LS dynamics during the perioperative period of the valve operation in comparison with the control group [at baseline, post-operative day 7 (POD7), and POD90]. The secondary endpoint was the correlation between LS and cardiologic parameters at each time point.

Baseline LS, ultrasonography, echocardiogram, and blood tests were performed in the case group within 7 days prior to the valve operation, all of which were repeated at POD7 and POD90. LS was collected from the control group during the three sequential perioperative time points (at baseline, POD7, and POD90). However, blood testing was conducted at baseline and at POD7, and ultrasonography and echocardiogram were performed only at baseline.

### Laboratory and cardiological assessments

Blood tests including complete blood count, aspartate aminotransferase, ALT, gamma-glutamyl transpeptidase (GGT), total bilirubin, blood urea nitrogen, creatinine, serum albumin, prothrombin time, and N-terminal pro brain natriuretic peptide (NT-pro BNP) were performed in all subjects.

Echocardiography was performed using a GE Vivid 7 ultrasound machine, and images were obtained from standard parasternal and apical views. Routine cardiologic parameters such as left ventricular ejection fraction (LVEF), left atrial volume (LAV), left atrial volume index (LAVI), left ventricle end systolic diameter (LVESD), left ventricle end diastolic diameter (LVEDD), and RVP were calculated according to the recommendations of the American Society of Echocardiography [15].

### Ultrasonographic examination and liver stiffness measurement

Upper abdominal scans were performed by two experienced hepatologists (>1,000 examinations) on all subjects to measure inferior vena cava (IVC) diameter and respiration-associated fluctuations in diameter. Evaluation of fatty liver was performed using the criteria of parenchymal brightness, liver to kidney contrast, and deep beam attenuation [16]. The presence of liver cirrhosis was checked by examining liver surface nodularity [17].

Immediately after a complete upper abdomen ultrasound examination, LS was measured according to a method reported previously [18]. Briefly, the LS measurement was performed on the right lobe of the liver through the intercostal space in patients lying in the dorsal decubitus position with the right arm in maximal abduction [19]. The operator located a liver portion that was at least 6 cm thick and free of large vascular structures, and pressed the probe button to commence the measurement. A single experienced technician (>10,000 examinations) performed the LS measurements. The success rate was calculated as the number of valid measurements divided by the total number of measurements. Interquartile range (IQR) was defined as an index of intrinsic variability of the LS measurement, corresponding to the LS interval containing 50% of the valid measurements between the 25th and 75th percentiles. LS is expressed in kPa, and the median value of successful measurements was representative. When LS showed an IQR to median value ratio of > 0.3, a success rate < 60% or < 10 valid measurements, it was regarded as invalid and was excluded from the final analysis [20]. Patients with LS < 5.5 kPa are regarded to have normal liver stiffness values [21], whereas patients with LS > 13.0 kPa are regarded as having an increased LS value compatible with liver cirrhosis or with increased hepatic congestion [4]. All ultrasonography and liver stiffness measurement operators were independent and blinded to the others' instrumental results and the patient clinical and laboratory data.

### Statistical analyses

The sample size of our study (*n*  =  31 in case group, *n*  =  11 in control group) with a significance level (alpha) of 0.050, achieved 85% power using a two-sided two-sample unequal-variance t-test. The sample size analysis was performed using the PASS software, version 12 (NCSS, LLC, Kaysville, UT, USA). Data are expressed as numbers (percent) or medians (range). Differences between patients in the valve operation and control groups were analyzed with the Mann–Whitney test or the chi-square test. LS differences within groups at baseline, POD7, and POD90 were evaluated by the Wilcoxon matched-pairs signed-rank sum test. Correlations between laboratory findings and LS were calculated in a bivariate analysis for non-parametric variables according to Spearman. Two sided *p*-values ≤ 0.05 were considered to indicate significance. The data analysis was performed using the SAS software, version 9.1 (SAS Institute, Cary, NC, USA).

## Results

### Baseline characteristics

All subjects in the case group had underlying TV regurgitation (either secondary or primary) of no less than a mild degree. Pulmonary hypertension (defined as mean pulmonary artery pressure > 25 mmHg) was present in 21 of 31 (77.4%) patients. The diagnosis and operative procedure for VHD were as follows: MV replacement due to moderate to severe rheumatic MV stenosis (*n*  =  10), MV replacement and TV annuloplasty due to moderate to severe rheumatic MV stenosis with secondary TV regurgitation (*n*  =  8), MV repair/replacement due to MV regurgitation caused by degenerative MV prolapse (*n*  =  5), MV repair/replacement and TV annuloplasty due to MV regurgitation caused by degenerative MV prolapse and secondary TV regurgitation (*n*  =  5), TV repair/replacement due to primary isolated TV regurgitation (*n*  =  3). Regarding cardiac function, 54.8% of patients belonged to New York Heart Association Functional Classification (NYHA) functional classification II, 35.5% belonged to NYHA class III, 6.5% to NYHA class I, and 3.2% to NYHA class IV (**Table 1**).

**Table 1:** Baseline and follow-up characteristics.

	Patients who underwent a valve operation	Patients who underwent a varicose vein operation	*P*
	(*n* = 31)	(*n* = 11)	
**Demographic variables**			
Age (years)	59 (36–79)	59 (45–76)	NS
Male gender	11 (35.5)	6 (54.5)	NS
Body mass index (kg/m^2^)	23.2 (19.8–24.3)	22.8 (19.5–26.0)	NS
Metabolic syndrome	8 (25.8)	4 (36.4)	NS
**Cardiac function**			
NYHA class I/II	2 (6.5)/17 (54.8)	–	
II/IV	11 (35.5)/1 (3.2)		
**Laboratory variables**			
AST (IU/L)			
Baseline	23.0 (12.0–144.0)	20.0 (12.0–32.0)	NS
POD 7	27.0 (14.0–116.0)	19.0 (14.0–43.0)	NS
POD 90	26.0 (14.0–36.0)	–	
ALT (IU/L)			
Baseline	17.0 (9.0–127.0)	16.0 (10.0–33.0)	NS
POD 7	20.0 (6.0–97.0)	21.0 (9.0–52.0)	NS
POD 90	18.0 (7.0–44.0)	–	
GGT (IU/L)			
Baseline	34.0 (10.0–207.0)	18.4 (9.0–73.0)	0.029
POD 7	67.0 (15.0–296.0)	21.0 (10.0–52.0)	0.001
POD 90	27.0 (12.0–74.0)	–	
Total bilirubin (mg/dL)			
Baseline	0.9 (0.3–3.1)	0.7 (0.4–3.0)	0.014
POD 7	0.6 (0.3–0.9)	0.6 (0.3–1.3)	NS
POD 90	0.8 (0.5–3.1)	–	
Baseline BUN (mg/dL)	15.1 (9.9–17.5)	14.6 (10.9–26.1)	NS
Baseline creatinine (mg/dL)	0.9 (0.7–1.3)	0.8 (0.7–1.2)	NS
**Ultrasonographic variables**			
Diameter of IVC (mm)			
Baseline	15.0 (5.6–19.8)	12.2 (4.8–17.5)	0.023
POD 7	13.5 (5.5–15.8)	–	
POD 90	14.7 (6.3–16.2)	–	
Respiration-associated fluctuation of the IVC diameter (mm)			
Baseline	2.2 (0.4–4.2)	2.4 (0.7–3.5)	NS
POD 7	2.5 (0.3–3.5)	–	
POD 90	2.6 (0.7–4.4)	–	

Variables are expressed as medians (range) or n (%).

NS, not significant (*P* > 0.05); NYHA, New York Heart Association; AST, aspartate aminotransferase; POD, postoperative day; ALT, alanine aminotransferase; GGT, gamma-glutamyl transpeptidase; BUN, blood urea nitrogen; IVC, inferior vena cava.

Baseline demographic, laboratory, and ultrasonographic characteristics are summarized in **Table 1**. The median age of patients in both the case and control groups was 59 years, and the prevalence of male gender in the case and control groups was 35.5% and 54.5%, respectively. GGT and total bilirubin were significantly higher in the case group than those in the control group (all *P* < 0.05). In all patients, baseline blood urea nitrogen and creatinine level were within normal range, and the median values of blood urea nitrogen and creatinine were statistically similar between the case and control groups (all *P*>0.05). At baseline, no patients showed evidence of hepatitis, fatty liver, or liver cirrhosis by ultrasonography. The diameter of the IVC was significantly greater in the case group than in the control group, but respiration-associated fluctuations in diameter did not differ between the groups (median 2.2 *vs.* 2.4 mm; *P*  =  0.204). Baseline LS and cardiologic characteristics, including NT-proBNP level, LVEF, LAV, LAVI, LVESD, LVEDD, and RVP are shown in **Table 2**. The median LS and NT-proBNP were significantly higher in the case group than in the control group (median 7.9 *vs*. 4.6 kPa, *P*  =  0.001 and median 585.0 *vs*. 21.6 pg/mL; *P* < 0.001; **Table 2**).

**Table 2:** Baseline and follow-up LS and cardiologic parameters.

	Patients who underwent a valve operation	Patients with varicose vein operation	*P*
	(*n* = 31)	(*n* = 11)	
**LS (kPa)**			
aseline	7.9 (3.9–73.5)	4.6 (3.3–6.8)	0.001
POD 7	8.4 (4.7–66.4)	4.4 (3.3–6.2)	< 0.001
POD 90	6.0 (4.0–66.4)	4.6 (3.6–5.4)	0.005
**Cardiologic parameters**			
NT-proBNP (pg/mL)			
Baseline	585.0 (76.3–6837.0)	21.6 (5.1–103.7)	< 0.001
POD 7	872.6 (384.5–8473.0)	24.0 (5.0–144.6)	< 0.001
POD 90	457.4 (49.3–2860.0)	28.1 (8.2–148.8)	< 0.001
LVEF (%)			
Baseline	66.0 (39.0–80.0)	–	–
POD 7	62.0 (34.0–78.0)	–	–
POD 90	63.0 (41.0–75.0)	-	–
LAV (ml)			
Baseline	136.1(53.2–550.7)	–	–
POD 7	85.9 (41.1–436.6)	–	–
POD 90	64.1 (36.4–388.6)	–	–
LAVI (ml/m^2^)			
Baseline	81.0 (19.0–353.0)	–	–
POD 7	53.0 (28.0–272.7)	–	–
POD 90	43.7 (21.7–247.5)	–	–
LVESD (mm)			
Baseline	34.0 (26.0–47.0)	–	–
POD 7	34.0 (27.0–53.0)	–	–
POD 90	32.0 (24.0–47.0)	–	–
LVEDD (mm)			
Baseline	49.0 (40.0–74.0)	–	–
POD 7	49.0 (40.0–66.0)	–	–
POD 90	48.0 (38.0–61.0)	–	–
RVP (mmHg)			
Baseline	45.0 (24.0–78.0)	–	–
POD 7	34.5 (20.0–71.0)	–	–
POD 90	28.0 (10.0–68.0)	–	–

Variables are expressed as medians (range) or n (%).

LS, liver stiffness; POD, post-operative day; LVEF, left ventricular ejection fraction; LAV, left atrial volume; LAVI, left atrial volume index; LVESD, left ventricle end systolic diameter; LVEDD, left ventricle end diastolic diameter; RVP, right ventricular pressure.

### Comparison between the case and control groups postoperatively

We compared the variables in the case and control groups postoperatively (**Table 1**). GGT level, which was significantly higher in the case group at baseline than that in the control group (median 34.0 *vs*. 18.4 IU/L; *P*  =  0.029), remained higher than in the control group at POD7 (median 67.0 *vs.* 21.0 IU/L; *P*  =  0.001). However, total bilirubin level, which was significantly higher in the case group than in the control group, became similar to that of the control group at POD7 (0.6 *vs*. 0.6 mg/dL; *P* > 0.05).

LS and NT-proBNP levels were consistently higher in the case group than in the control group from baseline to POD90 (all *P* < 0.05; **Table 2**).

### Postoperative changes in cardiologic parameters in the case group

Baseline NT-proBNP level significantly increased at POD7 in the case group (median 585.0 → 872.6 pg/mL, *P*  =  0.038) but decreased significantly at POD90 (median 872.6 → 457.4 pg/mL, *P*  =  0.048; **Table 2**). Although LVEF, LVESD, and LVEDD remained stable throughout the study period, RVP at POD7 and POD90 decreased significantly postoperatively compared to that at baseline RVP (median 45.0 → 34.5 mmHg, *P*  =  0.001 and median 45.0 → 28.0 mmHg, *P* < 0.001; **Table 2**). Similar findings were noted for LAV (median 136.1 → 85.9 mL, *P*  =  0.003 at POD7 and median 136.1 → 64.1 mL, *P*  =  0.001 at POD90; **Table 2**) and LAVI (81.0 → 53.0 mL/m^2^, *P*  =  0.014 at POD7 and 81.0 → 43.7 mL/m^2^, *P*  =  0.002 at POD90; **Table 2**) postoperatively.

### Postoperative changes in LS

**Figure 1:**
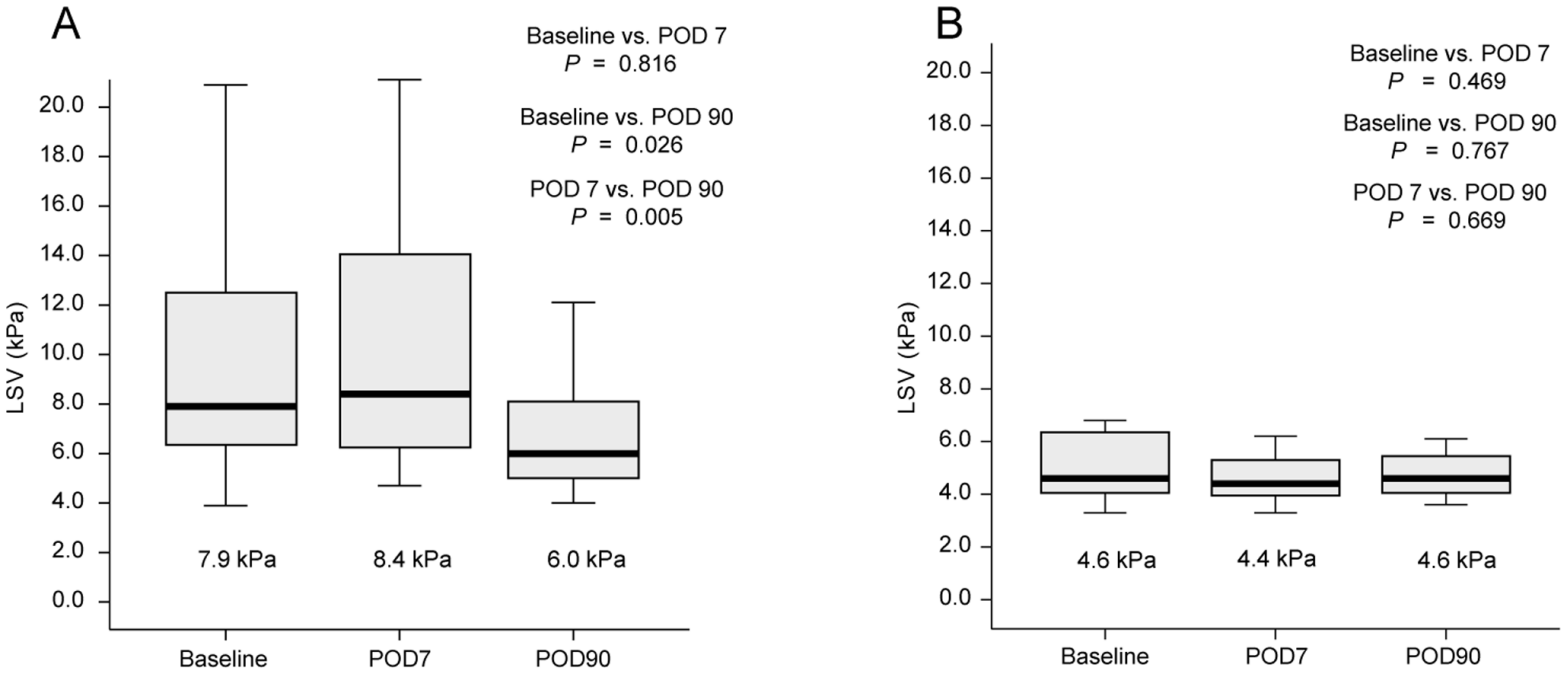
Liver stiffness at baseline, POD7, and POD90. (A) Patients with valve operation; LS increased minimally on POD7, but decreased significantly on POD90. (B) Patients with varicose vein operation; LS remained normal without significant interval changes during the study period. LS, liver stiffness; POD, post-operative day.

LS increased minimally at POD7 in the case group (median 7.9 → 8.4 kPa, *P*  =  0.816) but decreased significantly at POD90 (median 8.4 → 6.0 kPa, *P*  =  0.026; **Table 2** and **Fig. 1A**), whereas LS remained normal without significant interval changes during the study period (Table 2 and Fig. 1B) in the control group.

In case group, 25 (80.5%) patients showed elevated baseline LS (>5.5 kPa) [20]. Among them, eight (25.8%) had a LS in the range of liver cirrhosis or increased hepatic congestion (>13 kPa) [4]. Of these, seven patients experienced a drop in LS to < 13 kPa on POD90. Notably, there was one patient who showed LS of 73.5 kPa at baseline, and persistently high (73.5 → 66.4 → 66.4 kPa) LS during the study period. This was due to persistent deterioration of cardiac function in spite of the valve surgery. Among 17 (54.8%) patients showing a LS of 5.5–13 kPa in the case group, most patients (*n*  =  14) experienced a decrease in LS, and six of them showed normalization of LS on POD90. Six patients with normal baseline LS also showed normal LS on POD90.

In control group, 10 of 11 patients showed normal baseline LS (<5.5 kPa), and their LS during perioperative period changed within normal range. In one patient in the control group, baseline LS was 6.8 kPa, but it was normalized to 4.8 kPa on POD90.

### Correlation between LS and cardiologic parameters in the case group

**Figure 2:**
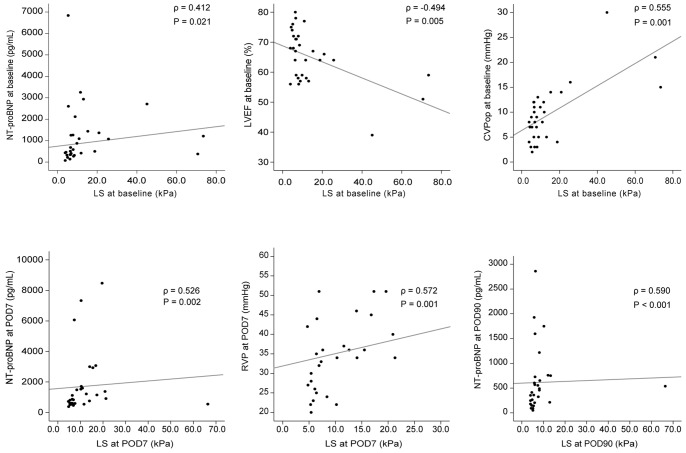
Correlation between liver stiffness and cardiologic parameters in patients with valve operation. LS was significantly correlated with NT-proBNP (ρ  =  0.412, *P*  =  0.021), LVEF (ρ  =  −0.494, *P*  =  0.005), and CVPop (ρ  =  0.555, *P*  =  0.001). LS was significantly correlated with NT-proBNP (ρ  =  0.526, *P*  =  0.002) and RVP (ρ  =  0.572, *P* =  0.001) on POD7. LS was significantly correlated with NT-proBNP (ρ  =  0.590, *P*  =  0.001) on POD90. LS, liver stiffness; NT-pro BNP, N-terminal pro brain natriuretic peptide; LVEF, left ventricular ejection fraction; CVPop, central venous pressure during the operation; POD, postoperative day; RVP, right ventricular pressure.

The correlations between LS and cardiologic parameters [(NT-proBNP, LVEF, LAV, LAVI, LVESD, LVEDD, RVP, and central venous pressure during the operation (CVPop)] at the same time points (baseline, POD7, POD90) were investigated. LS was significantly correlated with NT-proBNP (ρ  =  0.412, *P*  =  0.021), LVEF (ρ  =  −0.494, *P*  =  0.005), and CVPop (ρ  =  0.555, *P*  =  0.001) at baseline. LS was significantly correlated with NT-proBNP (ρ  =  0.526, *P*  =  0.002) and RVP on POD7 (ρ  =  0.572, *P*  =  0.001), whereas LS was significantly correlated with NT-proBNP on POD90 (ρ  =  0.590, *P*  =  0.001) (**Fig. 2**).

In case group, six patients who had shown baseline LS of 5.5–13 kPa experienced normalization of LS on POD90. In all of these patients, NT-proBNP values on POD90 reached to level of <300 pg/mL, which have a 98% negative predictive value for excluding acute HF [22]. The median NT-proBNP decreased from 349.3 (140.1–1264.0) pg/mL at baseline to 150.7 (49.3–265.0) pg/mL on POD90. LS showed significant correlation with NT-proBNP at baseline (ρ  =  0.403, *P*  =  0.025), and on POD90 (ρ  =  0.395, *P*  =  0.030).

## Discussion

In this pilot study, we demonstrated that increased baseline LS can be reversed after a successful operation in patients with VHD by measuring serial LS during the perioperative period. Notably, baseline and postoperative LS showed strong correlations with NT-proBNP, LVEF, CVP, and RVP, indicating the dynamic association between LS and restoration of cardiac function. Based on these results, the reduced role of TE for noninvasive monitoring of cardiac function in patients with VHD undergoing a valve operation might be revived.

A previous study by Millonig *et al*.[14] reported that LS is reversibly, directly, and tightly controlled by the intravasal venous pressure in experimental animals, regardless of basal LS assessed by the tissue matrix, and insisted that hemodynamic changes may strongly interfere with fibrosis assessments by TE. These results were validated by monitoring LS before and after diuretic treatment in ten patients with decompensated HF [14]. Another study by Colli *et al.* also reported about the significantly higher LS and NT-proBNP level in 27 patients with decompensated HF [23]. Compared to the study by Colli *et al.*, baseline median LS was lower in our study (7.9 *vs.* 8.8 kPa). It might be due to that patients included in our study are those who can afford the valve surgery, tended to be younger (age, mean ± standard deviation, 57 ± 12 *vs.* 79 ± 12 yrs) and to have preserved cardiac function (proportion of patients with NYHA class I and II, 61 *vs.* 15%) (**Table 1**). However, in both of above studies, detailed analyses of correlations between cardiologic parameters and LS to reveal the clear influence of perioperative hemodynamics on LS were not performed. This prompted us to systematically recruit patients with VHD who received cardiac catheterization during cardiac surgery and those who underwent varicose vein surgery as controls.

None of the patients in the case group showed any clinical symptoms of cardiac hepatopathy, such as hepatomegaly or ascites. However, liver-related laboratory abnormalities such as AST, ALT, GGT, and total bilirubin elevation were noted in some patients who underwent a valve operation. Moreover, the median values of GGT and bilirubin were significantly higher in the case group compared to the control group. The laboratory features of abnormal liver function mainly seem to reflect hepatic cholestasis due to passive congestion by increased systemic venous pressure, which are associated with right-sided HF. However, liver-related laboratory abnormalities in the control group are at most mild, and did not correlate with the cardiologic parameters.

Twenty-five of 31 patients in the case group had elevated baseline LS, and most (*n*  =  21, 84.0%) demonstrated a decreasing pattern of LS after surgical intervention. Of these, six patients further experienced normalization of LS (<5.5 kPa) on POD90. In contrast, LS of one patient was persistently high (> 65 kPa) during the study period. Although that patient received TAP due to severe TR, cardiac function did not improve with LS dynamics. These results suggest that TE can dynamically trace the changes in cardiac function after valve surgery. However, because baseline GGT, total bilirubin, IVC diameter, and NT-proBNP level in the case group were significantly higher than those in the control group, further studies should evaluate whether LS is a more sensitive surrogate for monitoring cardiac hemodynamics. Moreover, not all patients showed a decreasing pattern of LS or its normalization, although none of the participants had background chronic liver pathology. Further studies with a larger sample size are required to identify predictors of this discordant LS pattern phenomenon. In addition, whether decreasing LS patterns are related to long-term favorable outcomes of cardiac surgery using solid clinical endpoints, such as new cardiac event development or heart-related death, should be investigated.

The patient who showed persistently high (73.5 → 66.4 → 66.4 kPa) LS did not restore the cardiac function and died despite heart transplantation 7 months after the valve surgery. Although this patient did not show morphological sign of cirrhosis up to POD90, cardiac cirrhosis was detected on ultrasonography at 6 months after valve surgery with high LS value of 73.5 kPa at the same time. In this particular case, liver biopsy can be useful to better understand the underlying cause of liver damage whether it is due to the decompensated chronic HF or if there is any evidence of other preexisting chronic liver disease.

Correlations between cardiologic parameters and LS have been reported only rarely [13]. In our study, increased CVPop, which transmits backward to hepatic circulation, showed a marked correlation with baseline LS. Although we could not invasively monitor CVP postoperatively, we found that RVP on POD7 correlated highly with LS on POD7. Other than the animal study by Millonig *et al*. [14], this is the first human study to demonstrate that LS is directly ruled by backward pressure of the adjacent right heart during the perioperative period in patients with VHD. Because this backward pressure of the adjacent right heart is a continuous burden on the liver and can trigger cardiac fibrosis [24], patients with a cardiac problem whose LS remains high should be monitored carefully for the onset of liver fibrosis.

NT-pro BNP is an endogenous cardiac hormone synthesized in the cardiac ventricles as BNP, released as preproBNP, and then cleaved to produce NT-pro BNP [25], [26]. NT-pro BNP has not only been used as a diagnostic and prognostic marker for HF [27]–[30], but also reflects the clinical status of patients with VHD [31], [32]. The severity of regurgitation is directly correlated with NT-proBNP level in patients with mitral regurgitation [33], and, particularly, decreased NT-proBNP indicates reduced left atrial size, reversal of left ventricular remodeling, and improved symptoms for those who undergo successful mitral valve surgery [34]. In our case patients, LS was significantly correlated with NT-proBNP from baseline to POD90. Based on these results, we cautiously suggest that LS indirectly reflects cardiac hemodynamics and can be used to monitor cardiac function after valve surgery. However, further studies should investigate whether combined use of LS and NT-proBNP can monitor minute short-term postoperative cardiac outcomes as well as long-term outcomes such as heart-related mortality. Interestingly, NT-proBNP and LS values tended to increase slightly on POD7 compared to those at baseline. Although the main reason for this phenomenon is unclear, reactive pulmonary vasoconstriction and unrecovered liver congestion in immediate post-operative hyperdynamic status might be in part responsible.

We are aware of the limitations of our pilot study. Although we revealed correlations between LS and cardiologic parameters, this study was conducted for only a relatively short perioperative period (90 days) in a small population. Thus, the clinical implications of the changing patterns of LS, particularly for predicting long-term cardiologic outcomes, should be further explored in future studies. Secondly, we were not able to obtain liver biopsy specimen of the patients to investigate the relationship between the LS value and the pathology of cardiac hepatopathy.

In conclusion, LS can be overestimated in patients with VHD due to hepatic congestion, regardless of liver fibrosis severity. However, LS can be dynamically reversed during the perioperative period, reflecting the restoration of cardiac function after successful valve surgery. Further study with a larger sample size and longer follow-up period will reveal the clinical implications of LS measurements using TE and facilitate its expanded use in patients with VHD.
